# Aseptic necrosis of the femoral head after pregnancy: a case report

**DOI:** 10.11604/pamj.2016.24.195.7325

**Published:** 2016-07-07

**Authors:** Kawtar Nassar, Wafae Rachidi, Saadia Janani, Ouafa Mkinsi

**Affiliations:** 1 Rhumatology Department, Ibn Roch University Hospital, Casablanca, Morroco

**Keywords:** Aseptic necrosis, pregnancy, femoral head, risk factors

## Abstract

A documented case of beginning aseptic necrosis of the femoral head associated with pregnancy together with a review of the literature about this rare complication of pregnancy is presented. The known risk factors of osteonecrosis are; steroid use, alcoholism, organ transplantation, especially after kidney transplant or bone marrow transplantation bone, systemic lupus erythematosus, dyslipidemia especially hypertriglyceridemia, dysbaric decompression sickness, drepanocytosis and Gaucher's disease. Among the less established factors, we mention procoagulations abnormalities, HIV infection, chemotherapy. We report a case of osteonecrosis of femoral head after pregnancy.

## Introduction

Aseptic necrosis, known as avascular necrosis, ischemic necrosis, or osteonecrosis, is a pathological process caused by impaired blood supply to the affected bone and resulted in the death of osteocytes and bone marrow cells [1]. That induces demineralization, trabecular thinning, and subsequent collapse of the joint surface with fracture of subchondral bone. The first report was made by Pfeiffer in 1957 [2]. A clinical diagnosis is made according to the patient's symptoms, physical findings, and imaging results compatible with the disease. In our patient, all other predisposing factors, apart pregnancy have been excluded. Because until now few cases have been described, little is known about the evolution and treatment of this disease in association with pregnancy. We report a case.

## Patient and observation

**Figure 1:**
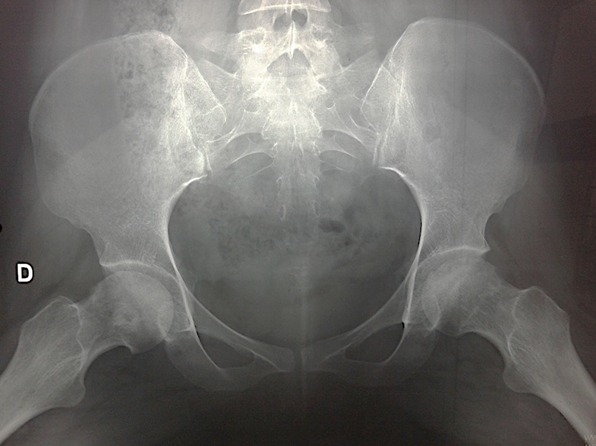
Left femoral heads subtle loss of sphericity

**Figure 2:**
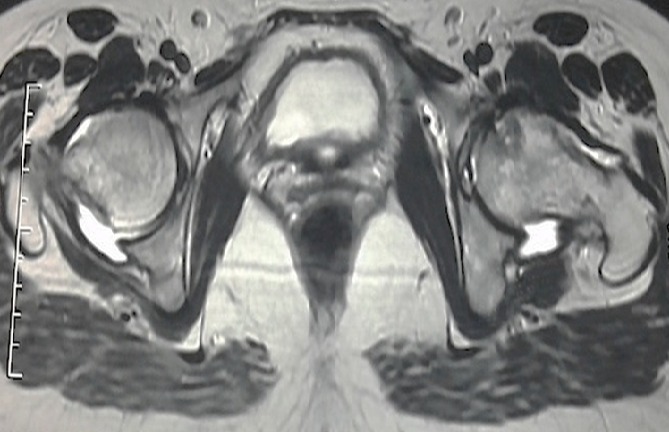
T2-weighted sequence MRI of the femoral heads showed bilateral joint effusion with focal lesion at the anterior part of the left hip

**Figure 3:**
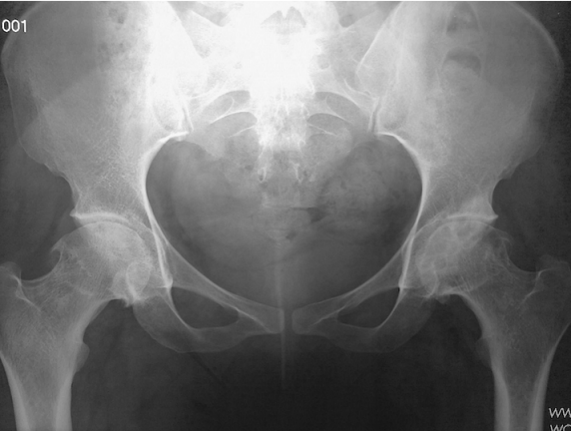
Prominent osteoarthritis, which is bilateral, with collapse of the articular surface and minimal loss of sphericity at the left femoral head

We report a case of 38 year old, housewife without past medical history. There was no history of steroid usage, alcohol drinking, or trauma. She presented in 2009, during the third trimester of pregnancy, mechanical pain in the left hip, persistent postpartum, causing walking discomfort. Blood analysis was normal in postpartum, and radiographs showed the femoral head subtle loss of sphericity (Figure 1). T2-weighted sequence MRI of the femoral heads showed bilateral joint effusion with focal lesion at the anterior part of the left hip (Figure 2). Symptomatic treatment was introduced to avoid complications and elective caesarean section was indicated. By the way, she consulted five years ago in our department for the same but bilateral symptoms, and pain became more severe at left without extra-articular sign. Physical examination revealed a walking limp, weight at 65 kg, and 167.5 cm for size. There was a pain in left hip flexion with limitation at 100 degrees. Key sign was positive with positive Patrick's (flexion abduction external rotation) tests especially at left hip. Radiographs showed prominent osteoarthritis, which is especially at right, with collapse of the articular surface and minimal loss of sphericity at the left femoral head, compared to the radio after pregnancy (Figure 3). Laboratory tests were normal (ESR at 26 mm/first hour, Rhumatoid Factor and antinuclear antibodies were negative, Hemoglobin electrophoresis showed; A1 at 97 and A2 at 3, antiphospholipid antibodies were negative, and normal lipidic analysis). There wasn't any abdominal ultrasound abnormities. Retained diagnosis was aseptic osteonecrosis of the left femoral head and secondary osteoarthritis at right by hyperpression. Core decompression surgical treatment was suggested, but the patient wished to receive medical treatment only to date (symptomatic treatment, oral bisphosphonate), associated to shockwaves. The outcome marked clinical improvement (walk without limping and pain), the radiographs are expected six months after starting treatment.

## Discussion

Pregnancy-associated femoral head osteonecrosis is rare. The first complaint is mostly unilateral, sudden or gradually increasing pain in the groin and radiating to the knee, thigh or back. Physical examination shows a painful limitation of active and passive movement of the joint, especially with rotation and less pronounced with abduction and flexion. Apart from traumatic forms, the exact causal link between supporting factors and aseptic necrosis occurrence is often uncertain which is only risk factors, clearly identified, rather than causes [3, 4]. A quarter to a third of aseptic necrosis is idiopathic, and occurs forty year-old men [5]. However, little is known about whether pregnancy is an etiological factor in femoral head osteonecrosis. Pregnancy is not considered as a risk factor of the disease or its recurrence in subsequent pregnancies. This association is a rare situation [6]. In 1999, Montella et al [7] reported the largest known series (13 cases), in all cases of which osteonecrosis affected the left hip and only 4 cases were bilateral. The pathogenetic mechanism of postpartum osteonecrosis of the femoral head are still controversial and probably multifactorial. Among mechanisms proposed, we found increased coagulability, mechanical stress, and impaired venous stasis. However, the high estrogen and progesterone production by the placenta can contribute to the development of osteonecrosis by fatty embolism. Also, the elevated non-protein bound cortisol and glucocorticoid activity of progesterone can be responsible [8]. The unbound maternal cortisol level is elevated to three times more among pregnant women than nonpregnant one. The pregnancy-related maternal parathyroid gland hyperplasia with high levels of parathyroid hormone may also contribute. Ischemia is frequent among pregnant women because pregnancy produces a hypercoagulable state, higher in the third trimester, with an overall monthly prevalence of 0.01 thrombotic events per 1,000 women [9]. Hypercoagulability may result in thromboembolism of vessels, which leads to vascular occlusion or venous congestion, and resulting ischemic necrosis of the bone. From the results of current studies, ovulation induction may be a cause which activate both coagulation and fibrinolytic systems [10, 11]. Mechanical stress induced by excessive weight gain during pregnancy may be another etiological factor for osteonecrosis of the femoral head [12]. Direct injury to the femoral joint or the artery in the round ligament by compression of the growing uterus or during a difficult delivery have been mentioned [13]. In our patient, the biochemical and coagulation parameters showed no abnormalities. There are six stages of aseptic necrosis, with typical clinical and histological signs in each stage. X-rays and conventional tomography become pathological only in stage 2, often several months after the onset of clinical symptoms, showing arc-like subchondral radiolucent areas, patchy lucent areas, sclerosis, a ′crescent′ sign and bone collapse [14]. Other techniques have to be used for early diagnosis: Bone scintigraphy, and resonance magnetic imaging (affected bone as early as 12-48 hours after the onset of the disease). The best way to treat the pregnant patient is not clear. The early diagnosis and advice are important. The prognosis after this conservative therapy seems to be good, although secondary degenerative or osteoarthritis changes eventually require surgical treatment at a later age.

## Conclusion

Aseptic osteonecrosis of femoral head associated to pregnancy is rare. The early diagnosis of this condition is important. Pain due to nerve compression will be partly relieved at rest. However, contrary to the pain of aseptic necrosis, there is an obvious exacerbation of the neuralgic pain during the night. The patients should be treated early and adequately to prevent secondary degenerative changes in the hip joints in these young women.
